# Benchmark dataset for the convex hull of 2D disks

**DOI:** 10.1016/j.dib.2019.104784

**Published:** 2019-11-12

**Authors:** Chanyoung Song, Joonghyun Ryu, Deok-Soo Kim

**Affiliations:** a School of Mechanical Engineering, Hanyang University, 222 Wangsimni-ro, Seongdong-gu, Seoul, South Korea; b Voronoi Diagram Research Center, Hanyang University, 222, Wangsimni-ro, Seongdong-gu, Seoul, South Korea; c HYU-HPSTAR-CIS Global High Pressure Research Center, Hanyang University, 222 Wangsimni-ro, Seongdong-gu, Seoul, South Korea

**Keywords:** Weighted points, Random disks, Circular container, QuickhullDisk, Incremental algorithm, Divide and conquer, Voronoi diagram

## Abstract

In this paper, we present a benchmark dataset which can be used to evaluate the algorithms to construct the convex hull of 2D disks. The dataset contains disk arrangements including general and extremely biased cases, which are generated by a C++ program. The dataset is related to an article: “QuickhullDisk: A Faster Convex Hull Algorithm for Disks” in which the QuickhullDisk algorithm is presented and compared to the incremental algorithm which was reported by Devillers and Golin in 1995 [1].

**Table:** Specifications Table

Subject	Computational Mathematics
Specific subject area	Computational geometry
Type of data	Text files (Each file represents an arrangement of disks)
How data were acquired	Generated by a C++ program on Intel® Core™ i7-7700 3.60GHz, 16.GB RAM with Window 10 operating system.
Data format	Raw
Parameters for data collection	1) Data type: RANDOM, ON-BNDRY, MIXED, ON-A-LINE. 2) Number of disks: a. RANDOM, ON-BNDRY, MIXED: 10K, 20K, …, 100K (K = 1000). b. ON-A-LINE: 1K, 2K, …, 20K. 3) Disk radius: a. RANDOM, ON-BNDRY, MIXED: Random in [1,10]. b. ON-A-LINE: 1.0. 4) Packing ratio: 0.1 (for RANDOM, ON-BNDRY, MIXED). 5) Container touching ratio: 0.1, 0.2, …, 1.0. (only for MIXED). 6) Distance between the boundaries of two neighbour disk: 0.5 (for ON-A-LINE).
Description of data collection	RANDOM: Disks are randomly generated within a circular container. ON-BNDRY: Disks are generated to touch a circular container from inside. MIXED: Disks are generated by a hybrid of RANDOM and ON-BNDRY. ON-A-LINE: Disks are generated in a linear fashion (sausage configuration).
Data source location	Voronoi Diagram Research Center, Hanyang University, Seoul, South Korea
Data accessibility	Title: The Source Code and Benchmark Dataset for QuickhullDisk, a Quickhull-like Algorithm for Constructing the Convex Hull of 2D Disks Direct URL to data: https://github.com/vdrc/The-Source-Code-and-Benchmark-Dataset-for-QuickhullDisk
Related research article	Nguyen Kieu Linh, Chanyoung Song, Joonghyun Ryu, Phan Thanh An, Nam-Dũng Hoang, Deok-Soo Kim, QuickhullDisk: A Faster Convex Hull Algorithm for Disks, Applied Mathematics and Computation, 363 (2019). https://doi.org/10.1016/j.amc.2019.124626.

**Table:** 

**Value of the Data** • The dataset can be used to evaluate the computation time of the algorithms to construct the convex hull of 2D disks. • The dataset can be useful to researchers in computational geometry community. • The dataset enables to compare the solution quality and robustness of the algorithms for the convex hull of 2D disks in diverse cases

## 1 Data

Convex hull is one of the most fundamental constructs in geometry and many algorithms to construct the convex hull have been extensively studied. Here, we present a benchmark dataset for the convex hull of 2D disks, which contains four types of disk arrangements spanning from general to extremely biased cases.

### 1.1 Four types of disk arrangements

1) RANDOM

A set RANDOM = {D_ij_|i, j = 1, 2, …, 10} where D_ij_ contains 10, 000 ∗ i random disks and represents a data file. Thus, there are ten different files with the same number of disks. We randomly placed the disks of D_ij_ within a circular container. The container center is at the origin which has a sufficiently large radius so that the packing ratio ρ is maintained approximately 0.1 unless otherwise stated. Note that ρ is the ratio of the union of the area of individual disks to the area of the container. In RANDOM (and the other two test sets ON-BNDRY and MIXED), each disk has a random radius r∈[1.0, 10.0], two disks may intersect each other, and one may include another.

2) ON-BNDRY

A set ON-BNDRY = {D_i_|i = 1, 2, …, 10} where D_i_ contains 10, 000 ∗ i disks touching a circular container from inside.

3) MIXED

A set generated by hybrid of RANDOM and ON-BNDRY: MIXED = {D_ij_|i, j = 1, 2, …, 10} where D_ij_ contains 1000 ∗ i ∗ j disks (out of 10, 000 ∗ j disks) touching a circular container from inside. The remaining 1000 ∗ (10 − i) ∗ j disks not touching the container are randomly positioned within the container. Hence, a container touching ratio γ is well defined as the ratio of the number of disks touching the container to the total number of the disks.

4) ON-A-LINE

A set of congruent disks centered on a line (known as a sausage configuration [2]): ON-A-LINE = {D_i_|i = 1, 2, …, 20} where D_i_ contains 1000 ∗ i disks on the linear grid from left to right. Each disk d ∈ D_i_ has a unit radius (1.0) and the distance between the boundaries of two neighbour disks is kept 0.5.

**Fig. 1:**
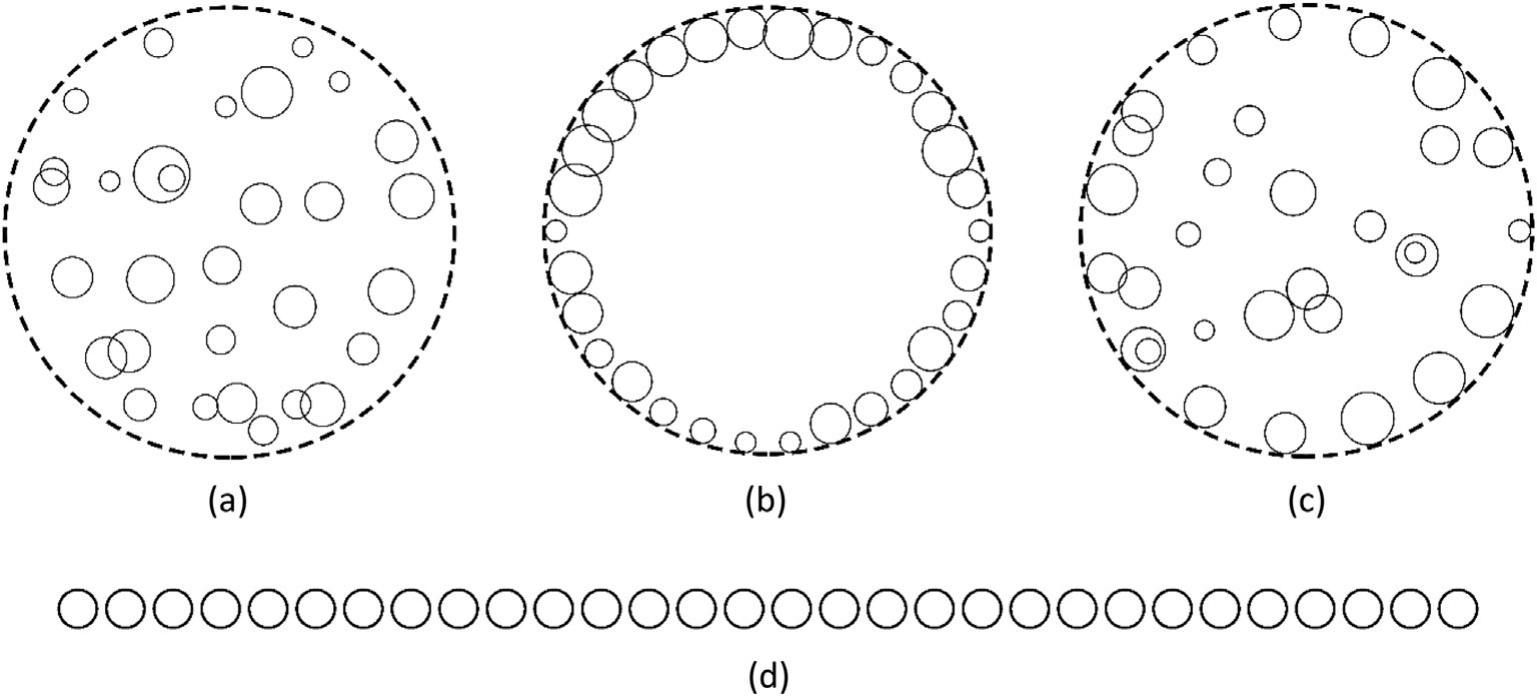
Four types of disk arrangements (30 disks for each) (a) RANDOM (b) ON-BNDRY (c) MIXED (d) ON-A-LINE.

Fig. 1 [1] shows examples of the four types of disk arrangements with reduced number of disks.

### 1.2 File format

For RANDOM, ON-BNDRY, ON-A-LINE, same file name convention is applied. Each file is named as ‘Nx.txt’ where ‘x’ denotes the total number of disks in the file. For example, a file ‘N10000.txt’ of RANDOM has 10,000 random disks. The file name of MIXED type includes the percentage of disks touching a container as well as total number of disks. A file ‘N10000_10.txt’ in MIXED includes 10% of the total 10,000 disks as disks touching a container (1000 disks).

**Fig. 2:**
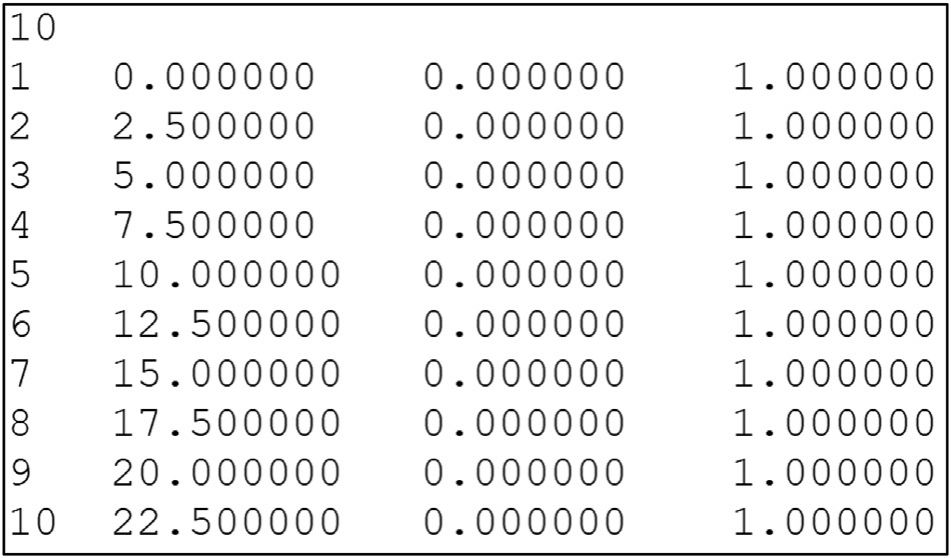
A data file corresponding to Table 1.

The file format is as shown in Table 1 where we added descriptions of row and column. Fig. 2 shows an example of a data file which corresponds to Table 1. The first line contains an integer denoting the number of disks in the file. Then, each following line contains the definition of each disk with 4 fields: disk id (integer); x- and y-coordinate of disk center (floating-point number); radius of disk (floating-point number). The floating-point number is represented up to six decimal places.

**Table 1:** An example for file format when ten disks are stored.

Number of disks: 10			
Id	X-coord. of center	Y-coord. of center	Radius
1	0.000000	0.000000	1.000000
2	2.500000	0.000000	1.000000
3	5.000000	0.000000	1.000000
4	7.500000	0.000000	1.000000
5	10.000000	0.000000	1.000000
6	12.500000	0.000000	1.000000
7	15.000000	0.000000	1.000000
8	17.500000	0.000000	1.000000
9	20.000000	0.000000	1.000000
10	22.500000	0.000000	1.000000

For RANDOM dataset, disks are stored in the generation order. For ON-BNDRY dataset, the rightmost disk touching a circular container appears first in a file and the other disks in counterclockwise (CCW) orientation follow. For MIXED dataset, the disks touching a container appears in a file like ON-BNDRY and then random disks follow. For ON-A-LINE dataset, the leftmost disk appears first and the others follow from left to right.

## 2 Experimental design, materials, and methods

We generate a benchmark dataset by a code written by C++ language in Window 10 operating system. The code's input parameters are data type (T), the number of disks (N), minimum radius (r_min_), maximum radius (r_max_) of disks, packing ratio (ρ), container touching ratio (γ) and distance between the boundaries of two neighbour disks (δ).

### 2.1 Disks in circular container

We fixed ρ, r_min_ and r_max_ as 0.1, 1.0 and 10.0, respectively. The code starts from generating a circular container C centered at the origin, whose radius R is calculated by the following (1) and (2).

(1) $$\text{S}=\left\{{\text{r}}_{\text{i}}\;\text{|}r_{min}+\left({\text{r}}_{max}-{\text{r}}_{min}\right)\text{*}\left(\frac{\text{i}}{\text{N}+1}\right)\text{,i}=1,2,\ldots ,\text{N}\right\}$$

(2) $$\text{ρ}=\frac{\cup \left(Area\;of\;Disks\right)}{\left(Container\;Area\right)}=\frac{\sum \pi r_{i}^{2}}{\pi R^{2}}$$

Note. The generated set S is used only for calculating the radius of container but not for random disks.

1) RANDOM

Each disk is randomly generated in an axis-aligned bounding box of the container C (i.e. random location in the box and random radius between 1.0 and 10.0). If a generated disk is completely in the circular container C, it is stored in a list. If not, a new disk with same radius is generated and tested again. Note that random number generator is used for generating random location and radius with current time as a seed unless otherwise stated.

2) ON-BNDRY

Each disk having random radius is generated to touch the container C from inside. The contact points to the container C are regularly spaced. The first generated disk is the rightmost extreme disk and the others follow in CCW order.

3) MIXED

First, disks touching container boudnary ∂C are generated like ON-BNDRY. Then, disks not touching ∂C are generated like RANDOM. A random disk could touch ∂C. To prevent this case, we choose a random disk within a shrunken container where the shrunken factor is 0.99.

Note. We confirmed that difference between computed packing ratio ρ of each data file and the fixed value (0.1) is less than 10^−3^. The contact points of disks to a container are different in 10^−6^ precision.

### 2.2 Disks in sausage configuration: ON-A-LINE

We set both r_min_ and r_max_ as 1.0. The first disk is generated to be centered at the origin and the other disks are generated to be centered on the x-axis where δ is kept 0.5.

Note that all the data files is freely available from our dataset repository [3] which can be used by QuickhullDisk programs in the same repository and Voronoi Diagram Research Center, Hanyang University (http://voronoi.hanyang.ac.kr/).
